# The role of differential delays in integrating transient visual and proprioceptive information

**DOI:** 10.3389/fpsyg.2014.00050

**Published:** 2014-02-03

**Authors:** Brendan D. Cameron, Cristina de la Malla, Joan López-Moliner

**Affiliations:** ^1^Vision and Control of Action Group, Departament de Psicologia Bàsica, Universitat de BarcelonaBarcelona, Spain; ^2^Institute for Brain, Cognition and Behaviour (IR3C)Barcelona, Spain

**Keywords:** position estimates, vision, proprioception, perceptual judgments, reaching

## Abstract

Many actions involve limb movements toward a target. Visual and proprioceptive estimates are available online, and by optimally combining (Ernst and Banks, 2002) both modalities during the movement, the system can increase the precision of the hand estimate. The notion that both sensory modalities are integrated is also motivated by the intuition that we do not consciously perceive any discrepancy between the felt and seen hand's positions. This coherence as a result of integration does not necessarily imply realignment between the two modalities (Smeets et al., 2006). For example, the two estimates (visual and proprioceptive) might be different without either of them (e.g., proprioception) ever being adjusted after recovering the other (e.g., vision). The implication that the felt and seen positions might be different has a temporal analog. Because the actual feedback from the hand at a given instantaneous position reaches brain areas at different times for proprioception and vision (shorter for proprioception), the corresponding instantaneous unisensory position estimates will be different, with the proprioceptive one being ahead of the visual one. Based on the assumption that the system integrates optimally and online the available evidence from both senses, we introduce a temporal mechanism that explains the reported overestimation of hand positions when vision is occluded for active and passive movements (Gritsenko et al., 2007) without the need to resort to initial feedforward estimates (Wolpert et al., 1995). We set up hypotheses to test the validity of the model, and we contrast simulation-based predictions with empirical data.

## Introduction

The more rapidly the hand moves, the harder it becomes for the sensorimotor system to localize it in real time. We might have the intuition that we can directly see and feel where our hand is at any time, but sensory feedback takes time to reach the central nervous system, so each sensory sample lags the hand's real location. This is an important problem for the sensorimotor system, because motor commands are noisy [especially when the limb is moving quickly (van Beers et al., 2004)] and there is likely to be some error in the initial motor plan. If a correction is to be applied, the sensorimotor system needs to acquire a reliable estimate of the hand's location. In other words, to effectively correct the hand in flight, the sensorimotor system must know where the hand will be relative to the target when the correction occurs. It has been suggested that such an estimate is achieved by optimally integrating vision, proprioception, and a copy of the original motor command (efference copy) (Desmurget and Grafton, 2000). An internal model of the motor system could, theoretically, use the integrated information to forecast the hand's location.

An important piece of evidence for the putative role of efference copy in real-time hand localization is the tendency for participants to overestimate the current location of their unseen moving hand (Dassonville, 1995; Wolpert et al., 1995). The temporal pattern of overestimation is consistent with the involvement of an internal forward model (Wolpert et al., 1995). Here we re-examine the overestimation phenomenon, and we propose an alternate explanation, one based on the optimal integration of differentially-weighted visual and proprioceptive location estimates when the hand moves in the dark.

### Estimating the current location of the unseen moving hand

Where do people perceive their moving hand? One way to measure this is to provide a visual, tactile, or auditory cue during motion of the hand and then have participants retrospectively report where the hand was when the cue was presented (Dassonville, 1995; Gritsenko et al., 2007). Alternatively, a “stop” signal can be provided during the movement, after which participants report the location of their stopped hand (Wolpert et al., 1995; Gritsenko et al., 2007). These methods tend to show that participants overestimate how far their hand has traveled; however, the effect is not universal, as we will discuss shortly.

In an influential study testing perception of the unseen moving hand, Wolpert et al. (1995) observed a hand position overestimate of 0.5–0.9 cm that was present throughout the measured range of time points (movement durations of 0.5–2.5 s). In that study, participants first viewed their static hand for 2 s, after which vision was occluded, and then participants generated slow planar movements to the left or right of the start location, stopping the movement as soon as a tone was played. Movement distance, dictated by when the tone was played, ranged from 0 to 30 cm. After stopping their movements, participants used a trackball to position a visual marker over the perceived location of their unseen hand. The pattern of perceptual reports—an increasing then decreasing overestimate as movement time increased from 0.5 to 2.5 s—was consistent with a state estimation process where efference copy (combined with the initial estimate of the limb) is initially weighted more heavily than sensory information. Wolpert et al. proposed that as the movement progresses, the reliability of the prediction based on the initial state estimate decreases and the contribution of sensory information to the following state estimates accordingly increases. This shift in weighting from the forward model prediction to the sensory-based estimate was modeled with a Kalman filter, where the weights assigned to the prediction-based estimate and the sensory estimate are dependent on their relative accuracies. Wolpert et al. argued that a pattern of increasing then decreasing overestimation could not be explained by a purely sensory model. We will outline later how a sensory processing model may, in fact, be able to account for such a pattern.

Dassonville (1995) also observed a position overestimate of the moving hand. In Dassonville's study, participants began each trial with their arm extended and pointing toward an LED. A second LED was then illuminated, and participants rapidly moved their hand to point at the second LED. Prior to each trial room lights were illuminated, providing participants full vision of their limb and surroundings, but each trial was conducted in the dark, such that only the LEDs were visible. A tactile stimulus was applied to the index finger, either before, during, or after completion of the movement. After completing the pointing movement to the target LED, participants used the same limb to reach back to the location at which they sensed the application of the tactile stimulus. Dassonville observed that, on average, participants reported a location that was approximately 100 ms farther along the trajectory of the initial reach than where the tactile stimulus was applied. In spatial terms, the overestimate ranged from 0 to 30 cm, depending on the stage of the reach at which the tactile stimulus was applied. Interestingly, participants reported overestimation of the stimulus position even when it was presented just before the onset of the reaching movement. Dassonville argued that consistent overestimation of hand position during movement may be caused by the sensory processing delay for the tactile stimulus. By the time the participant registers the stimulus, their internal representation of the moving limb (presumably aligned with the actual position of the moving limb) has moved beyond the position at which the stimulus was applied. Accordingly, the participant reports a position that is positively biased. However, this explanation is difficult to reconcile with the pattern of results observed by Gritsenko et al. (2007), described next.

Gritsenko et al. (2007) also examined perception of limb position during movement, but they examined not only active movements, in which participants move their own arms, but also passive movements, in which participants' arms are moved for them. Gritsenko et al.'s goal was to test whether a position overestimate would occur in the absence of active movement; that is, would participants report a position overestimate during passive movement, when no efference copy is present? In Gritsenko et al.'s study, participants executed/experienced planar, single-joint 140° movements of their lower arm, which was occluded for the entirety of the testing session. In one condition, the participant's task was to remember the location of their moving hand at the time that a sensory cue was presented, and then to execute a return movement to that location, as in Dassonville (1995). Gritsenko et al. observed very similar results for active and passive exposure: Participants tended to overestimate limb position early in the movement (approximately the first 60° of the movement), but they then underestimated it later in the movement (approximately the last 60° of the movement). Gritsenko et al. suggested that this pattern could be explained by a Bayesian process, in which the unreliability of sensory estimates during motion of the limb led to a heavy weighting of the prior (previously experienced elbow angles in this case). They speculated that this prior might have been biased toward the midpoint of the elbow's range of motion, which would then have caused early cues to be overestimated and later cues to be underestimated.

In another condition of Gritsenko et al.'s (2007) study, the participant's task was to stop their movement when the cue was presented and then report the location of the stopped hand, as in Wolpert et al. (1995). Gritsenko et al. again observed little difference between active and passive exposure; however, participants underestimated the distance traveled by the arm at all tested angles, a result that contrasts with the consistent overestimation effect observed by Wolpert et al. (1995). Several methodological differences exist between Gritsenko et al.'s and Wolpert et al.'s stop tasks, so we do not know which difference is responsible for the conflicting results. There was no visual information regarding hand start location in Gritsenko et al., information that was available in both Wolpert et al. (1995) and Dassonville (1995); furthermore, Gritsenko et al. studied a single-joint movement, whereas Wolpert et al. (1995) and Dassonville (1995) studied multi-joint movements. Either or both of these factors may be responsible for the different effects. However, for our purposes the important finding from Gritsenko et al. (2007) is the close correspondence of the position estimates from the active and passive exposures. This finding suggests that some mechanism that is independent of efference copy might explain position misestimation during reaching.

None of the studies we have described here included a comparison condition in which vision of the reaching hand was available during the reach. Presumably, the researchers assumed that vision would allow for highly accurate position estimates and so they did not include full-vision conditions. However, if efference copy contributes to early position estimates, its effect on the movement should be present regardless of the type of sensory information (visual or proprioceptive) that is available. If, on the other hand, misestimation of the reaching hand actually depends on removing real-time vision (as is implicit in the studies discussed above), a mechanism for misestimation of the moving hand that relies on intersensory re-weighting (instead of prediction-to-sensory re-weighting), is worth considering.

## A temporal mechanism based on differential delays

We propose that some of the perceptual overestimation effects that have previously been reported can be explained with a temporal mechanism. Our temporal sensory-integration hypothesis is based on two premises: (1) that proprioceptive feedback is processed more quickly than visual feedback, and (2) that the integrated estimate of the reaching hand is more strongly influenced by the more reliable unisensory estimate (Ernst and Banks, 2002; Smeets et al., 2006). Accordingly, when people reach in the dark, the integrated estimate of their hand shifts toward the more reliable (and temporally leading) proprioceptive estimate. After presenting evidence for the differential delays between proprioception and vision, we will provide a basic rationale for how such a mechanism would work.

### Evidence for differential delays

#### Visual delays

It takes at least 40 ms for a visual stimulus to reach V1. This relatively long latency (compared to other transduction latencies, such as the ones in auditory processing) is mainly due to the time that photoreceptors need to encode information. About 120 ms after visual stimulation, activation can be found in most cortical areas, and leads to conscious visual experience (e.g., Raiguel et al., 1989; Nowak et al., 1995; Lamme et al., 1998; Lamme, 2000, 2003; Lamme and Roelfsma, 2000). In total, the time that one needs to react to a visual stimulus has been estimated to be approximately150–200 ms, as will be described below (e.g., Brenner and Smeets, 2003; Barnett-Cowan and Harris, 2009).

One common task to measure differential delays and to compare them across modalities is the simple reaction time (RT) task, in which the experimenter measures the time that it takes to react to a stimulus of a determined sensory modality. In RT tasks, the difference between the sensory modalities provides us with an approximate value of the lag that one of the sensory modalities has to have with respect to another one in order for the participant to perceive them as simultaneous. From RT results, the time needed to react to a visual stimuli is about 150–220 ms (e.g., Brenner and Smeets, 2003; Barnett-Cowan and Harris, 2009), although this value can vary depending on factors such as the intensity of stimulation (e.g., Schiefer et al., 2001). However, one must take into account that RT is a behavioral measure and so the values provided do not only contain the signal processing time but also the time needed to react. To deal with the “extra time” added by the motor output, some authors have used neurophysiological techniques like ERPs (e.g., Rugg and Coles, 1995; Thorpe et al., 1996) to measure how long the processing period takes. By using this method Thorpe et al. (1996) concluded that highly demanding tasks involving visual image processing can be solved in 150 ms or even less.

Another way of measuring delays in the visual system is by looking at response times to target location changes. By perturbing the target's position one can measure how long it takes to correct an ongoing movement (e.g., Georgopoulos et al., 1981; Soechting and Lacquaniti, 1983; Prablanc and Martin, 1992; Brenner and Smeets, 1997, 2003; Veerman et al., 2008; Oostwoud-Wijdenes et al., 2011). Brenner and Smeets (1997) found that it takes about 400 ms to react (to start moving) to a visual stimulus subjects had to hit (this is the result of processing the visual stimuli, planning the hitting movement, and initiating the response). When the target is displaced during a movement, several factors influence how quickly the movement can be adjusted toward the target's new position. One of these factors may be the uncertainty about the direction of the possible position change. Soechting and Lacquaniti (1983), using double-step paradigms in which the direction of the change was known, reported that the time that it takes to modify trajectories was similar to reaction times toward the first stimulus and of the order of 110 ms. The time to respond may increase if the direction of the target change is not known. Boulinguez and Nougier (1999), for instance, showed a faster correction time (191 ms) for a 75% predictable location than for a 50% and 25% predictable location (213 and 211 ms, respectively) (cf. Cameron et al., 2013).

The timing of the perturbation can also affect the latencies of the corrections. Liu and Todorov (2007) found that the latency to correct an ongoing movement is of about 100 ms independently of the timing of the perturbation. Although this result is in accordance with others (e.g., Gritsenko et al., 2009; Oostwoud-Wijdenes et al., 2011), there are authors that have suggested that the closer to the end of the movement the perturbation takes place, the longer the latency of the correction (e.g., Reichenbach et al., 2009). Other factors affecting how quickly subjects can respond to a target position change are the attributes of the target: faster responses are observed toward targets defined by orientation, size or luminance than by color, texture or shape (e.g., Veerman et al., 2008).

There has also been some research on responses to visual perturbations of the position of the hand or of a cursor or a tool representing the hand's position (e.g., Saunders and Knill, 2003, 2004, 2005; Franklin and Wolpert, 2008; Proteau et al., 2009; Brière and Proteau, 2011). The most common situation in the cursor-jump experiments is that subjects have to move a cursor that represents the hand position toward a target and at some point the cursor jumps so that the trajectory of the movement has to be corrected. The reported latencies of the corrections for cursor jumps are about 140–160 ms (Saunders and Knill, 2003; Franklin and Wolpert, 2008; Veyrat-Masson et al., 2010), slightly larger than the ones for target jumps.

#### Proprioceptive delays

Proprioception, which provides information related to body posture, is derived from receptors in skin, muscles, tendons, and joints. Accordingly, proprioceptive transmission time to the brain depends on the body part from which the signal originates. For this, and other reasons it is not easy to arrive at a precise estimate of proprioceptive processing times, but we outline some data in the following paragraphs that allow for an approximation.

In non-human primates, the time needed for afferent signals from proprioception to reach brain areas has been estimated to be as little as about 30 ms (Fetz et al., 1980; Soso and Fetz, 1980; Evarts and Fromm, 1981). In a study comparing reaction times to a visual stimulus and to a kinaesthetic one in humans, Flanders and Cordo (1989) found that it took approximately 250 ms to react to a visual stimulus and only 150 ms to react to a kinaesthetic one. In that study subjects had to modulate the left elbow torque in response to a stimulus that could be presented either visually or kinaesthetically. For the visual task, subjects saw the stimulus moving for 70 ms and had to increase or decrease the left elbow torque in a determined direction depending on the final position of the stimulus. For the kinaesthetic task, subjects' right elbow was rotated and they had to increase or decrease the left elbow torque in response to how the right elbow was rotated. In another study with an easier task, Flanders et al. (1986) reported smaller values but in the same direction (110 ms for kinaesthetic information and 190 for visual information). Shorter latencies were reported in Johansson and Westling (1987) who found compensatory responses after 75 ms in response to feedback from the skin receptors in a grip task.

Alary et al. (1998) recorded ERPs when passively moving the right index finger of healthy subjects. The shorter latencies they found were of about 56 and 32 ms for the right and the left index finger respectively in P1 (parietal areas) and of 115 and 96 ms respectively for N1 (frontal areas). Similarly, Mima et al. (1996) also used passive movement of the index finger and evoked potentials and reported the earliest cortical latencies in P1 of 34.6 ms and N1 at 44.8 ms. Seiss et al. (2002) showed that the latency values obtained for both flexion and extension were similar, and of about 90 ms in the N90 component. Factors such as the kind of stimulation (or the device used to create it) or the stimulated area could be responsible for the different values obtained in studies using ERP measures.

Although it is difficult to come up with a reliable estimate of the differential delays, from the data presented above we can estimate sensory delays of about 50–60 ms for proprioception and of about 100–120 ms for vision. So, in conclusion, we can say that proprioception leads vision by approximately 40–50 ms.

### Rationale of the mechanism

**Figure 1:**
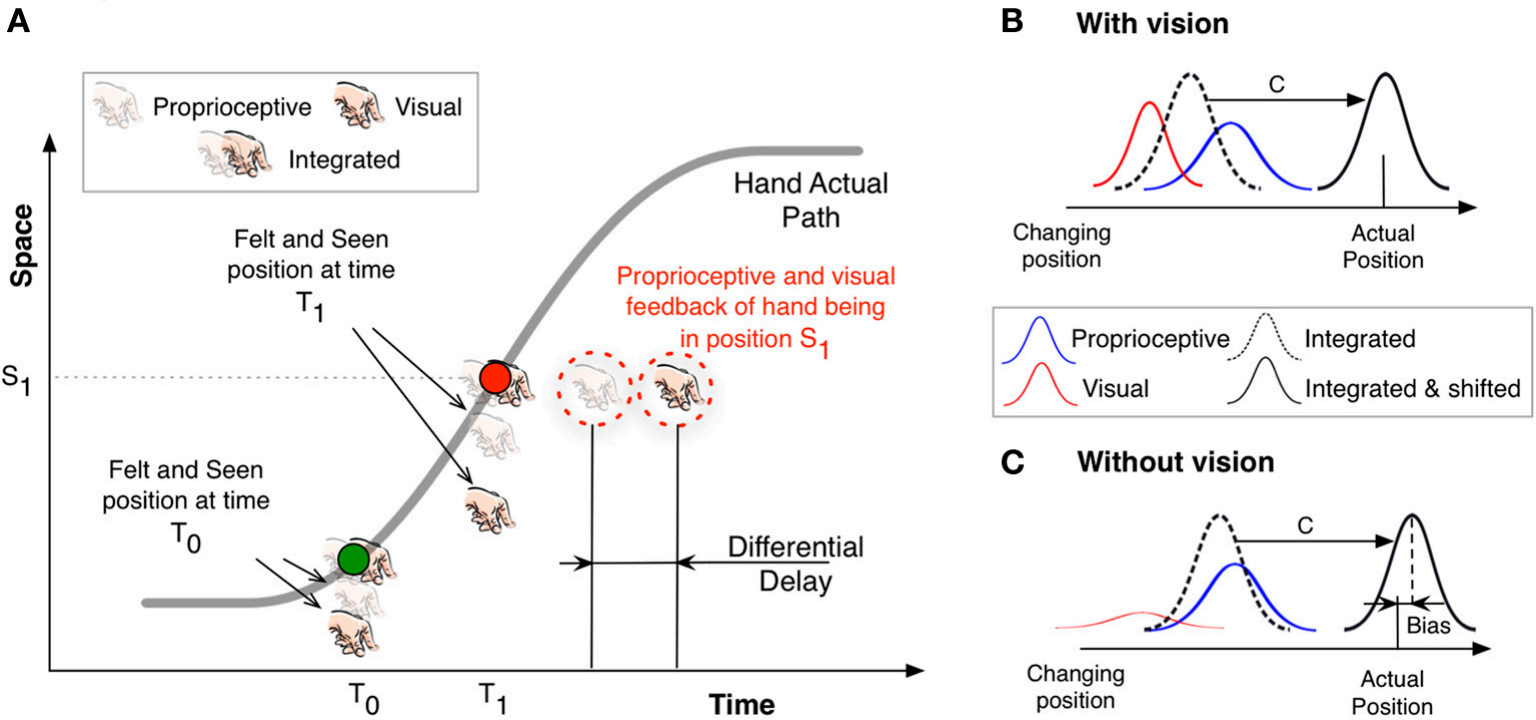
**Sketch to illustrate our rationale. (A)** The gray curve denotes the actual path traveled by a hand: the changing position in one dimensional space is plotted against time. The slope of the curve at any given point describes the tangential velocity at this particular time. The green and red points therefore correspond to moments at which the hand moves slowly early in the path (green) and when it moves at the highest speed half way to the target (red). See text for details. **(B)** Sketch of the position estimate based on integrated information. The dashed curve denotes the integrated estimate based on visual feedback (red) and proprioceptive feedback (blue). A constant shift is assumed to correct for the sensory delays. **(C)** The same as **(B)** but without visual information. The visual estimate (red) has a larger uncertainty.

Figure 1 illustrates the main features of the proposed mechanism by showing the changing position of a hand along a one-dimensional path (gray curve) through time. The slope of this curve thus denotes the velocity of the hand. Two colored points indicate samples at two timepoints (*T*_0_ and *T*_1_) along the trajectory. The green dot denotes the instant position at *T*_0_ in the early part of the path, after the hand has just started to move and the speed is not yet very high. The red dot represents the instant position of the hand at time *T*_1_, when the hand is moving at peak velocity. Assuming the presence of differential delays, in accordance with the evidence reported above, the main idea is that the unisensory positional feedback of the hand at each of these two instant positions will reach the corresponding unisensory brain areas at different times. For example, when the hand moves slowly at time *T*_0_ the corresponding instant position will be acquired by visual areas later than by proprioceptive areas. As a consequence, the online visual estimate lags the proprioceptive one. This differential latency in reaching the corresponding areas also manifests in a spatial shift between the visual and proprioceptive position estimates. This situation is represented by the vertical distance between the visual and proprioceptive feedback around the green dot in Figure 1. Because this spatial discrepancy results from a temporal difference, we predict that the felt and seen position will be sensed as being the furthest apart when the hand moves at peak velocity (time *T*_1_), as illustrated by the separation between proprioceptive and visual estimates around the red dot. From this point on, the spatial separation of the two unisensory position estimates will decrease.

### Integrated information and delayed feedback

Relying only on available re-afferent signals to update changing positions of the limbs will necessarily lead to delayed actions or overreaching to static targets. We therefore have to assume some kind of adjustment when we integrate both unisensory estimates of position. In Figure 1A the integrated percept is ahead of the two unisensory ones and aligned with the actual hand position. This is an important problem in perception mainly caused by the neural transmission times in the sensory systems and has led to the persistent question of whether the perceived position of a moving object lags its “real position” (e.g., Cavanagh, 1997; Krekelberg and Lappe, 2001). Neural delays are present at both sensory and motor stages and, similarly to the internal models proposed to compensate for motor delays, additional compensatory sensory mechanisms have also been put forward (e.g., Nijhawan, 2008) to extrapolate the position of moving objects at the perceptual level. Most evidence for such a sensory mechanism comes from the flash-lag phenomenon (Nijhawan, 1994; Linares et al., 2007), in which a flashed object is perceived to lag a physically aligned moving object. This fundamental problem would apply to both vision and proprioception. In Figure 1A we have aligned the integrated percept of the hand with the actual hand position. This situation is also reproduced in Figure 1B, which illustrates one position sample of a moving hand. The integrated estimate is shifted (magnitude C) ahead to compensate for the neural delay. One can think of these perceptual mechanisms that correct for sensory delays as calibration mechanisms that would shift the corresponding integrated percept in space. However, what is important for our explanation is the relative difference between the visual and proprioceptive estimates irrespective of any compensation mechanism (e.g., extrapolation) to correct for these delays. When visual information is not present the visual estimate of the changing position is no longer reliable, but the system will still integrate, we assume, the information according to a maximum likelihood principle (Ernst and Banks, 2002). This will cause the integrated estimate to be shifted toward the proprioceptive position, which is now (without vision) more reliable. As a consequence, and after the compensation mechanism that shifts the position estimate to compensate for neural delays in feedback processing, the felt position of the hand is further ahead (Figure 1C) relative to when vision is available.

## Prediction of perceptual bias from transient proprioceptive information

The perceptual bias for the moving limb tends to be in the direction of motion, that is, the limb is felt ahead of the actual position. Importantly, this bias does not appear to be constant along the whole limb trajectory but rather increases in the first part of the movement and decreases afterwards (e.g., Wolpert et al., 1995) and sometimes a bias in the opposite direction (behind the actual position) has been reported during the last part of the movement (e.g., Gritsenko et al., 2007).

**Figure 2:**
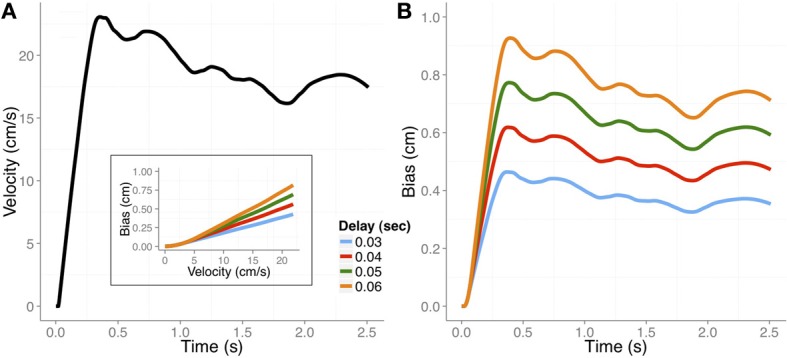
**(A)** Velocity profile of a hand movement in a pursuit task (adapted from Rodríguez-Herreros and López-Moliner, 2008). **(B)** The expected perceptual bias across time would be determined by the velocity profile and the differential delay (color coded) between vision and proprioception. Inset: The expected bias as a function of tangential velocity for the four possible differential delays used in the simulation. See text for more details.

It is important to keep in mind that this bias is relative to the actual position of the hand and it is often implicitly assumed that there would be no bias when the transient position was judged with vision of the hand. To our knowledge, however, no evidence has been reported for this. In Figure 2 we plot the basic predictions regarding the perceptual bias in judging a transient position of the hand at the time of an external cue.

Our model, which is based on differential delays, makes strong predictions about the trend of the bias along the movement. Specifically, the amount of bias is mainly determined by the velocity of the limb at the time the probe or cue signals the moment of the judgment. This prediction is largely consistent with the observation in both Dassonville (1995) and Gritsenko et al. (2007) that position overestimates increase with increasing velocity. Unfortunately, the studies reporting perceptual biases of the unseen limb provide limited information about instantaneous limb velocity. In addition, the velocity profiles in those studies are not always easy to infer from the performed limb movements. Furthermore, the movements in some of the studies were not very fast, with movement times typically longer than 1.5 s. Slower movements may have been used to facilitate tracking of the felt position of the limb. In Figure 2A we plot the velocity profile from a pursuit task (Rodríguez-Herreros and López-Moliner, 2008) which is similar to the speed of the movements used in some of the studies addressing the perceptual bias during movement (Wolpert et al., 1995; Gritsenko et al., 2007). The velocity profiles determine the expected biases for the different delays, which are shown in Figure 2B. We outline in the next section how we computed these biases.

### Simulations of perceptual overestimation

We assume that the integrated estimate of hand position is aligned with the actual hand position. As a corollary of this assumption, the resultant percept is shifted to the proprioceptive one when vision is absent. The scenario depicted in Figure 1A would be equivalent to having an integrated estimate in which the variance for vision is very large, but with residual visual memory preventing it from reaching infinite values. To demonstrate the possible effects of such a mechanism, we use a real movement from a pursuit task (Rodríguez-Herreros and López-Moliner, 2008) so that we have the velocity *v*_*t*_ and actual position *p*_*t*_ of the hand across time *t*. Because the integrated position (*p*^*vp*^_*t*_) is aligned in time with the actual one (*p*^*vp*^_*t*_ = *p*_*t*_), the integrated position can be expressed as:

(1) $$p_{t}^{vp}=w_{v}\times p_{t}^{v}+w_{p}\times p_{t}^{p}$$

However, due to the different visual and proprioceptive delays, the unisensory estimates of positions *p*^*v*^_*t*_and *p*^*p*^_*t*_ will lag behind the actual position (*p*_*t*_) to different extents (the visual position will lag more). *p*^*v*^_*t*_ would correspond with the visual estimate of the position at time *T*_1_ in Figure 1A and *p*^*p*^_*t*_ would correspond with the proprioceptive estimate at the same time. *w*_*v*_ and *w*_*p*_ are the weights given to the visual and proprioceptive estimates and are detailed below.

In order to compute the bias one could, therefore, obtain the unisensory estimates for vision and proprioception by finding earlier positions within a movement, such that the proprioceptive estimate would correspond to the actual position some time steps prior to the current position, and the visual estimate would correspond to an even earlier position. However, because the bias only depends on the differential delay between vision and proprioception, for simplicity we assumed no delay for proprioception in our simulation. Accordingly, we included in Equation 1 delayed positions for vision and updated positions for proprioception. We used 30, 40, 50, and 60 ms of delay (proprioception leading vision), values that include lower and upper bounds for the differential delays reported in the literature (discussed above).

The weights for vision and proprioception depended on the reliability of each modality for localizing the hand. We set *w*_*v*_ = (1/σ^2^_*v*_)/(1/σ^2^_*v*_ + 1/σ^2^_*p*_) and *w*_*p*_ = (1/σ^2^_*p*_)/(1/σ^2^_*v*_ + 1/σ^2^_*p*_) where σ^2^ denotes the variance of the modality. We used variances of 1 and 0.56 cm^2^ for proprioception and vision, respectively, which are very similar to corresponding uncertainties of position estimates reported in previous studies (van Beers et al., 1999; de la Malla and López-Moliner, 2012). The actual perceptual bias was finally computed as the difference between the estimated position when there is no vision and the integrated position when there is full vision. (We assumed an infinite variance for the visual estimate when vision was absent, so the no-vision estimate is essentially equal to the proprioceptive estimate.) Figure 2B shows the predicted bias obtained from the same velocity profile shown in Figure 2A for the different delays.

The model captures the main trend reported in many of the studies: the bias is larger in the early part of the movement and decreases by the end. As we think the bias is caused by the differential delays, its magnitude will follow the velocity profile of the movement. For example, in Wolpert et al. (1995) the bias reaches a maximum of about 1 cm after 1 second of movement and drops afterwards. In Figure 2, there is a higher acceleration in the early part of the movement due to the fact that subjects had to catch up with the moving target after they started to move. In spite of these differences, the magnitude of the predicted bias caused by the differential delays is not very different from that reported in Wolpert et al. (1995). The inset of Figure 2 illustrates the relation between the predicted bias and the tangential speed of the limb at the time of the judgments. The bias as a function of the tangential speed could be approximated by a linear function whose slope would be very close to the differential delay between vision and proprioception.

One important feature of the explanation based on the heavy weighting of efference copy in the early part of the trajectory (Wolpert et al., 1995) is that the bias should vanish when the movement is passive. As mentioned earlier, Gritsenko et al. (2007) found the same pattern of estimation error for active and passive movements. Our model, which is based on differential sensory delays makes the same predictions for both active and passive movements.

Interestingly, Gritsenko et al. (2007) found a difference in the reported bias between fast and slow movements in the same direction that we would predict from our model. A larger bias was observed for fast movements which is consistent with the bias having originated, at least in part, from the differential delays. However, they also report a bias in the opposite direction (judgments behind the actual position) by the end of the movement. Our model cannot explain this finding, but a confound could be present during the last tested positions in their study. They used eight angle positions to obtain the judgments and the cue changed color to signal the transient position at which subjects had to make the judgment. The cue was uniformly distributed across the different angles, so that as the movement unfolded the cue expectancy was progressively increasing. Therefore, the expectancy was higher for larger angles (late part) than for smaller ones (early part). This attentional factor could have accelerated the processing of the late cues relative to early ones, thereby reducing the bias at late cues.

### Fitting perceptual overestimation data

**Figure 3:**
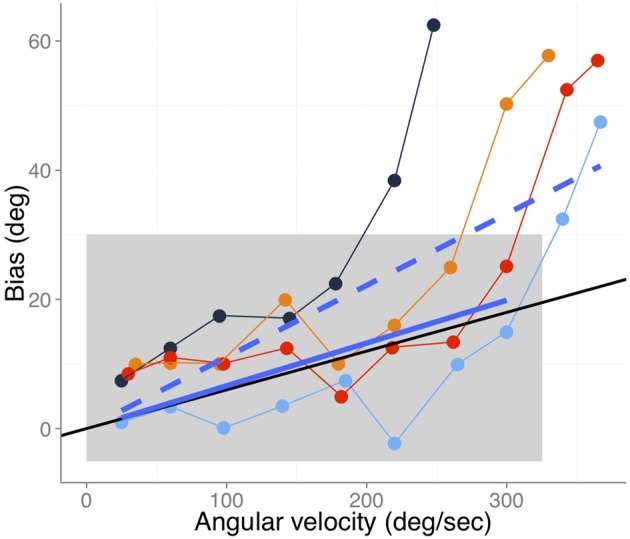
**Bias as a function of angular velocity adapted from Gritsenko et al. (2007), (Figure 7A) for the different active movement conditions.** Different colors code the angles at which the probe was shown while moving the arm (60, 75, 90, and 105° in blue, black, orange, and red respectively). The black line denotes the expected bias assuming a differential delay of 60 ms between vision and proprioception. The blue solid line denotes the best fit (slope 0.066 s and zero intercept) including the data points that fall within the gray rectangle. The dashed solid line (slope 0.133 s and zero intercept) denotes the fit to all data points.

Gritsenko et al. (2007) provide information about the hand's velocity at the moment of the probe; therefore, we are in the position to illustrate to what extent our model can predict part of this study's data. In Figure 3 we reproduce the data points from the active movement conditions shown in Figure 7A of Gritsenko et al. (2007). In this study the authors found no significant differences between active and passive movements. For the data that we show here, subjects extended their arms from 40° flexion between the upper arm and forearm to full extension (180°). At some designated angle during the movement a cue (change of color plus a beep) was shown as a mnemonic cue. After completing the movement, subjects had to report the perceived position of the hand at the time of the cue. Figure 3 shows data for four of the tested angles (60, 75, 90, and 105°) which are color-coded.

At a first glance one can see the strong dependency between the bias and the speed of the hand. However, the differential delay account predicts a linear dependency between hand speed at the time of the probe and the reported bias. Therefore, our explanation cannot fully account for the data pattern shown in Figure 3. Nevertheless, note that the bias can go as high as 60° and seems certainly larger than biases of about 1 cm like those reported in Wolpert et al. (1995), which are in the prediction range of our differential delays hypothesis. One can also notice that there is an initial linear trend for all the conditions shown in Figure 3 (data points within the gray rectangle). We fitted a linear model with only a single parameter (slope only and zero intercept) to this set of points. The blue solid line represents this linear fit which yielded a slope of 0.066 s, very close to the black solid line that denotes the predicted bias given a differential delay of 60 ms (near the upper bound of our estimate of the differential delay). This model accounts for 70 percent of the variability (*R*^2^ = 0.71). Although it is clear that the data do not behave linearly across the entire velocity range, we also provide the fit to all the data points for information purposes only. The slope for this fitted line is 0.13 s (dashed line in Figure 3), which is well beyond the upper bound of estimated differential delays between vision and proprioception. Some other factors must cause the exponential increase of the biases. Another important point is that data points within the linear part scatter quite a lot around the linear fit. Part of this variability appears to be explained by the angle at the time of the probe, with smaller angles showing larger biases.

In sum, our differential delay hypothesis can account fairly well for the linear trends shown in the overestimation biases reported in Gritsenko et al. (2007).

## Prediction of “under-reaching” to static targets

If people tend to overestimate the real-time position of their moving hand, it makes sense that they would also tend to under-reach the target: if the moving hand is felt to be closer to a target than it really is, movements should tend to be halted prematurely. However, it is not clear whether the perceptual and the motor phenomena have common underlying mechanisms. At first glance there are some clear differences. When participants are instructed to make perceptual reports, the system is encouraged to monitor the changing position of the limb, and this goal constrains the speed of the limb in order to meet the task requirements. On the other hand, reaching to static targets does not necessarily involve monitoring the changing position of the limb. For very fast reaching movements, it is unlikely that the system keeps track of the changing position. Instead, for movement times less than 200 ms, open-loop strategies probably control the hand. Yet, it is possible that for longer movement durations the under-reaching reported in some studies could in part be explained by the temporal mechanisms we are proposing. In the next section we explore this possibility. In order to do so, we conducted simulations to obtain some indicative magnitudes of the bias based on the differential delays.

### Simulations of under-reaching

**Figure 4:**
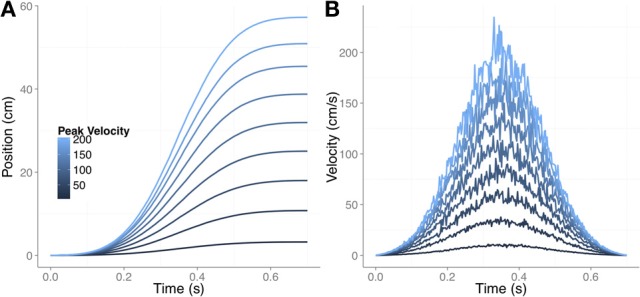
**The nine different movements used in the simulation of under-reaching bias. (A)** Changing position of the finger for the different movements. The movement time was always 0.7 s and the peak velocity varied from 10 cm/s (the slowest movement) until 200 cm/s (the fastest movement). **(B)** The corresponding velocity profiles with signal-dependent noise. Note that in **(A)** the noise is not noticeable after integrating the tangential velocity.

We started with 9 movements with bell-shaped velocity profiles, all of which had equal movement times but different peak velocities. Figure 4A shows the one-dimensional trajectories for the different movements. Figure 4B reproduces the velocity profile (noisy version) for each movement. For each movement we simulated 1500 trajectories as follows. In each of the 1500 iterations we first obtained a noisy version of the velocity profile. The noise was signal-dependent Gaussian noise (Harris and Wolpert, 1998), and Figure 4B shows one example for each type of movement. We then integrated the information to obtain the varying time series of the actual hand position for each trial. From the actual trajectory we then derived the feedback-delayed proprioceptive and visual estimates for each time step as follows.

We assumed that the initial position (before any movement) of the integrated hand estimate was aligned with the actual hand position (Smeets et al., 2006). In each trial of the simulation, the initial felt and seen positions of the hand were randomly drawn from a Gaussian distribution with a *SD* of 1 cm and 0.75 cm for proprioception and vision, respectively, centered around the actual position of the hand. These values correspond to the variances used before. Once we had the unisensory estimates of the initial positions we computed the delayed unisensory running estimates based on the previously obtained velocity profile of the actual movement. This produced two time series of changing position, one for vision and another for proprioception, with the only difference being the starting position, which was drawn at random.

**Figure 5:**
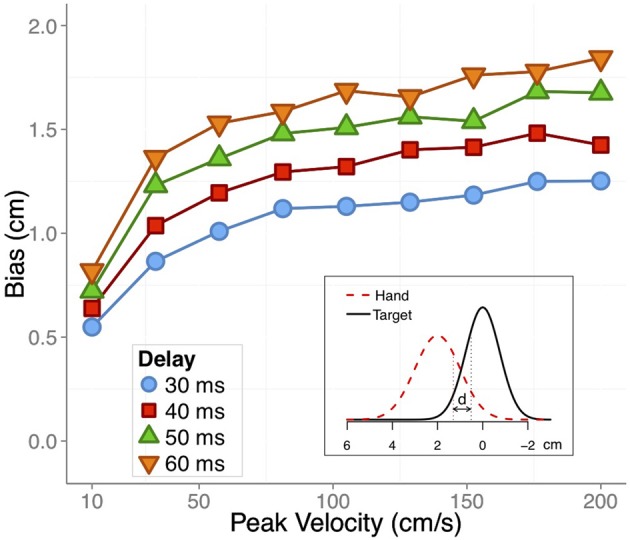
**The expected values for a bias in under-reaching static visual targets with the unseen hand as a function of peak velocity in simulated movements**. Different colors and symbols denote differential delays between visual and proprioceptive feedback. Inset: illustration of the estimation of the felt position of the hand (dashed red Gaussian) and the estimation of the static target (solid black Gaussian). A running distance (denoted by d) between both Gaussian was computed to determine the final end point based on unisensory estimates of the hand. See text for details.

We then computed the integrated estimate by using Equation 1 as we did before. At each time step in which we computed the integrated estimate of position, the proprioceptive estimate corresponded to the same time step, but the visual estimate corresponded to a past position. The amount that the visual estimate lagged the proprioceptive estimate depended on the size of the differential delay. For each simulated trial and movement we used the same set of differential delays between vision and proprioception that we used before (30, 40, 50, and 60 ms, with proprioception leading vision). In order to get a measure of the bias, we compared the no-vision estimate (assuming infinite variance for visual reliability) and the integrated full-vision estimate. The running bias would then be maximum at peak velocity. To stop the movement we computed an error signal between the running estimate and the target position, which was defined as the final position of a template movement (in this case, final position when vision and proprioception are both available). When the error was less than a threshold we stopped the movement. The inset in Figure 5 illustrates the threshold mechanism that we used. We computed a distance between two Gaussians, one representing the felt (or integrated) position of the hand (red-dashed Gaussian) and the other denoting the estimated target position (black-solid Gaussian). In the case of the no-vision estimate, as shown in the inset, the *SD* was set to 1 cm while for the integrated condition the *SD* was 0.6 cm (variance of 0.36 cm^2^, derived from optimally combining proprioception and vision). The *SD* for the target localization was 0.75 cm (which results in a variance of 0.56 cm^2^, the same used in Figure 2). The final end point was obtained by using the following expression:

(2) $$Q(p=0.75,\;\mu ,\;\sigma )-Q(p=0.25,\;\mu_{T},\;\sigma_{T})<1$$

where Q is the inverse Gaussian cumulative distribution function; μ and μ*_*T*_* are the no-vision or full-vision position estimate and the target position estimate, respectively. σ and σ *_*T*_* are the corresponding uncertainties (SD) for limb and target estimates. When the difference between quantile 0.75 of the limb position and quantile 0.25 of the target position (d in the inset of Figure 5) was less than 1 cm the movement was stopped. In this way we obtained the full-vision and no-vision endpoints, and could compute the relative difference between both as a measure of the bias. Figure 5 shows this bias as a function of the peak velocity of the different nine movements that we simulated in Figure 4. The biases are shown for the four different differential delays used to compute the running position estimates for vision and proprioception.

Note that, as before, the reported simulated bias is independent of the compensation mechanisms that shifts the integrated percept of the hand to account for the transmission delays.

### Comparison with previously reported under-reaching

The majority of studies of endpoint bias, to our knowledge, report an under-reach bias during targeted reaching (e.g., Soechting and Flanders, 1989; Chieffi et al., 1999; Engelbrecht et al., 2003; Diedrichsen et al., 2004; Elliott et al., 2004; Krigolson and Heath, 2004; Oliveira et al., 2005), but some have reported an over-reach bias (e.g., Lönn et al., 2000; Westwood et al., 2003), and some studies suggest that the presence and magnitude of an under-reach bias depends on the delay between target occlusion and the onset of the reach (Westwood et al., 2003; Krigolson and Heath, 2004). It is not clear why the recency of target information influences the magnitude of under-reaching, but it may have more to do with trial-to-trial error minimization than with the real-time estimate of the hand. Indeed, motor optimization is likely to contribute significantly to endpoint biases. Under-reaching a target has potential benefits for system efficiency, as it protects against movement reversals, which can incur time and energy costs to the performer (Engelbrecht et al., 2003; Elliott et al., 2004; Oliveira et al., 2005). It is likely, therefore, that under-reach biases are in part caused by strategic modulation of the feedforward impulse as a way to minimize costly error corrections (Engelbrecht et al., 2003).

The sensory-integration hypothesis that we have presented here is consistent with an under-reaching behavior, but our hypothesis can only explain the portion of the bias that is related to the real-time estimate of the moving hand. Unfortunately, this putative sensory portion of the under-reach bias has not been isolated from feedforward contributions in previous research, so the model-based estimate that we provided here may not be directly comparable to previously reported under-reach magnitudes. Matters are further complicated by the different protocols used in previous studies of movement under-reaching; often, participants are directly immersed in a no-feedback reach environment, with no prior calibration of motor commands. (Our model assumes prior calibration of reaching, such that the feedforward component is properly calibrated to target distance.) This absence of calibration in some studies might explain, for instance, dramatic under-reaching for some open-loop tasks [up to 15 cm (Soechting and Flanders, 1989)], and overreaching in others (e.g., Khan and Franks, 2000; Westwood et al., 2003). Without motor calibration, different reach conditions, such as unconstrained whole-arm reaching to remembered targets in Soechting and Flanders (1989) and 1-dimensional planar movements constrained by a manipulandum in Khan and Franks (2000), may produce distinct biases that are unrelated to the online estimation phenomenon we address with our model. Future studies will be needed to test whether our model can account for any under-reach effects.

## Alternative explanations for position overestimation during reaching

### Biases caused by efference copy

In the introduction we described Wolpert et al.'s (1995) explanation for position overestimation during reaching, which proposed that the early state estimates of the moving limb are dominated by an efference copy-based prediction. Wolpert et al.'s model provides a nice fit for their data; however, it also relies on the assumption that the motor system has access to both an efference copy and an internal forward model. In contrast, our model assumes neither efference copy nor forward modeling to produce a similar pattern of increasing and decreasing overestimation as a movement progresses, and in this sense it is the simpler model. However, we did make the assumption that a perceptual shift of the position estimate compensates for sensory delays (Figure 1). This predictive processing is more ‘general-purpose’ than the efference copy-based predictive processing employed in Wolpert et al.'s model, in that the same perceptual mechanisms that allow someone to predict the upcoming location of any moving stimulus, despite sensory processing delays, could also be employed for forward-shifting the estimated location of the moving hand. Whether or not our assumption of sensory compensation is simpler than Wolpert et al.'s assumption of motor-based prediction is arguable. However, it is important to note that our proposed compensatory shift does not have any influence on the *pattern* of overestimation produced by our model, whereas for Wolpert et al. (1995) motor prediction is integral to the pattern of overestimation. In fact, our model's predictions about differences between visual closed- and open-loop position estimates do not rely on any assumptions about compensatory shifting of the sensory estimates. Perhaps the best reason for favoring our model over an efference copy-based one, though, is that our scan account for the presence of limb position overestimation during both active and passive movements (Gritsenko et al., 2007). Moreover, our model can explain the velocity-dependence of the overestimation effect in both active and passive movements (Gritsenko et al., 2007).

One shortcoming of our model, however, is that it does not explain the underestimation performance that has been reported for later parts of a movement (Gritsenko et al., 2007). At this point, we cannot be sure if the late position underestimation is an artefact of the experimental protocol employed by Gritsenko et al. and if the effect is, therefore, independent of the estimation process we are attempting to explain. This, however, puts us in the tenuous position of potentially cherry-picking effects from Gritsenko et al. that support our model, such as the similar behaviors for passive and active exposures. That being said, we believe that the similar *patterns* of performance observed in the passive and active conditions is a more important effect than the actual size and direction of the estimation bias, which is likely to be sensitive to the specific protocol employed. Furthermore, because there was no comparison between vision and no-vision conditions in the Gritsenko et al. study (the comparison, strictly speaking, that our model is designed to describe), we cannot know the extent to which the underestimation effect at late cue times is inconsistent with our model.

In the end, we cannot state with certainty that our model is superior to an efference copy model for explaining position misestimation during movement. At the very least, however, we have presented a plausible sensory-driven mechanism for the misestimation phenomenon. Future comparisons between visual closed- and open-loop position estimation will test the quality of our model.

### Biases in the proprioceptive map

One possibility that we have not yet addressed is that position overestimates are not related to movement per se, but rather to differences between visual and proprioceptive spatial maps. When the hand moves away from the body (as it does for most reaching movements), the hand may occupy locations at which the proprioceptively-sensed position is different from, and farther away from the body than, the visual one. Wilson et al. (2010), for instance, have shown that the right hand tends to be felt as though it is farther to the right than it really is. Thus, if participants make a rightward reach with their unseen right hand, their hand estimate might lead the real hand, producing perceptual position overestimates and, potentially, under-reach performance. (Position overestimates during leftward reaching with the right hand (e.g., Wolpert et al., 1995) would be harder to explain.)

While such proprioceptive biases may contribute to the overestimation phenomenon during reaching, we suspect that they do not account for all of it. Gritsenko et al. (2007), for instance, showed a speed-dependent overestimation effect, which suggests that motion of the hand does have an influence on the position estimate that is independent of the hand's current location relative to the body. Furthermore, Gritsenko et al. (2007) showed that reports of the stopped (i.e., static) hand exhibited a different pattern (one that did not meaningfully vary as a function of spatial location) than reports of the remembered location of the moving hand. Future experiments that directly compare static position reports with spatially-matched motion reports would help to clarify the contribution of a participant's proprioceptive map to the misperception of his or her moving limb.

### Switching between visual and proprioceptive estimates of the hand and the possible effects of realignment

We have assumed that visual and proprioceptive estimates of limb position are integrated and that integration depends on the relative reliabilities of each estimate (van Beers et al., 1999; Ernst and Banks, 2002; Smeets et al., 2006). We have also assumed that the estimates are independent of each other, i.e., that one sense does not realign the other one (Smeets et al., 2006).

It is possible, however, that sensory estimates are not integrated. Rather, it may be that when vision is available it dominates position sense, and when vision is absent proprioception dominates position sense. This would not affect the direction of the bias that we have modeled, but it would increase the size of the bias. The predicted bias would be equal to the difference between the proprioceptive estimate (reaching in the dark) and the delayed visual estimate (reaching in the light), rather than the difference between the proprioceptive estimate (reaching in the dark with infinite variance for the visual estimate) and the integrated estimate (reaching in the light with weighted estimates).

It is also possible that the proprioceptive estimate is spatially realigned by vision (e.g., Cressman and Henriques, 2009). The effects of such spatial realignment on the running estimate of the hand as it moves in the dark would depend on the rate and direction of the deterioration of the alignment when people reach without vision. If the proprioceptive estimate remained stable after removal of vision, the direction of effector misestimation would be similar to what we have proposed here. If the proprioceptive estimate decayed, the effect on position estimation in the dark would depend on the direction of the decay.

Perhaps a more pertinent consideration is whether the proprioceptive estimate is *temporally* realigned by vision when visual feedback is available (that is, whether the sensorimotor system delays proprioceptive feedback in order to sync it with slower visual feedback). The effect of such alignment on position estimates following visual occlusion would depend on the rate of its decay in the dark. If temporal alignment decayed quickly, we would expect position overestimates to arise after only a few movements in the dark. If the decay occurred slowly, the overestimation bias would develop more gradually. As long as the decay was toward the baseline processing speed for proprioception (i.e., faster than vision), one should observe an overestimation bias. However, the rate at which the bias developed might differ from what we have modeled here.

### Biases in the localization of moving objects

Judgments about the location of moving objects at the time of a probe usually result in reported positions that are too far along their path (e.g., Brenner and Smeets, 2000; Whitney et al., 2000; Alais and Burr, 2003; Ögmen et al., 2004; Brenner et al., 2006). This is the very same pattern obtained for transient positions of the unseen moving limb with the only difference being that in the former case the target is an external object. This similarity raises the question of whether the phenomenon addressed here is caused by the same mechanisms as the biases generally reported for moving objects. One needs a time of interest at which to judge the position of a moving object and this is usually signaled by using flashes or tones. However, there is still much debate about the mechanism and functionality of this bias that is consistent with an extrapolation of motion. The idea that this bias in the direction of motion compensates for sensory delays motivates one of the explanations of this phenomena and the flash-lag effect (Nijhawan, 1994). By the time a physically aligned flash is detected (as a time marker), the moving object will have moved to a new position causing the spatial misalignment. This explanation is not very different than the one proposed by Dassonville (1995) to account for the positive bias in the estimate of the moving hand.

Interestingly, Nijhawan and Kirschfeld (2003) reported a flash-lag effect between a flash and a rod moved with an unseen wrist. Subjects perceived a spatial misalignment between the rod and the flash. Note that this type of judgment involves comparing the position of the controlled rod relative to the cue, as in the typical flash-lag task, rather than ascertaining the position of a moving object at the time of the probe. The bias reported in Nijhawan and Kirschfeld (2003) is, however, in the same direction as the ones discussed in this article: subjects perceived the flash lagging the tip of the rod. In this study the flash or probe was presented when the rod was moving at the maximum velocity. Although the value was not reported, the average speed of the movement was 63.8 cm/s, which means that the maximum speed was higher then this value. The magnitude of the flash-lag was between 6 and 8 cm which is, admittedly, larger than would be predicted from the differential delays between proprioception and vision. There is, however, a clear difference between this study and the others. In Nijhawan and Kirschfeld (2003) the judgment relied on always comparing visual information and not a proprioceptive location at the time of a probe. Like the model outlined here, the flash-lag effect also has a clear dependency on velocity of the moving object (e.g., López-Moliner and Linares, 2006). Carefully designed experiments will, therefore, be needed to address the question of whether the bias when judging proprioceptive positions is actually a consequence of compensatory mechanisms for proprioceptive delays.

## Future directions

Our hypothesis that differential delays between vision and proprioception contribute to position overestimation provides a new perspective on how the sensorimotor system monitors the real-time location of a moving limb. If our hypothesis is correct, it might imply that efference copy is either not incorporated into the real time estimate of the limb or that it is incorporated in an un-biasing way.

Our model makes specific predictions about how the estimate of the limb should be influenced by different movement speeds and, while these predictions are consistent with previously-reported overestimation effects, future experiments are needed that specifically examine position estimates as a function of the hand's instantaneous velocity, while controlling for both cue expectancy and the spatial location of the cue relative to the participant.

We also recommend some control procedures for future investigations of real-time position estimation: (1) probing position estimates in both visual open-loop *and* closed-loop conditions, and (2) probing position estimates when the hand is moving *and* when the hand is static (or, alternatively, changing the start location and direction of reaches across trials, such that they span the workspace and thereby control for any effects of the location of the cue/target relative to the body). We also suggest that more agreement among studies might be obtained if researchers ensure that participants' reaches remain calibrated across trials. Such calibration might be achieved, for instance, by randomly inserting, among test trials, motor calibration trials in which performance feedback is provided.

Finally, we hope that future studies will examine the relationship between real-time perceptual estimates of the reaching limb and goal-directed reach performance. While it is tempting to assume that perceptual position overestimation is directly related to an under-reaching bias, we are not aware of any studies that have tested this link.

### Conflict of interest statement

The authors declare that the research was conducted in the absence of any commercial or financial relationships that could be construed as a potential conflict of interest.

## References

[B1] Alais D. Burr D. (2003). The “flash-lag” effect occurs in audition and cross-modally. Curr. Biol. 13, 59–63 10.1016/S0960-9822(02)01402-1 12526746

[B2] Alary F. Doyon B. Loubinoux I. Carel C. Boulanouar K. Ranjeva J. P. (1998). Event-related potentials elicited by passive movements in humans: characterization, source analysis, and comparison to fMRI. Neuroimage 8, 377–390 10.1006/nimg.1998.0377 9811556

[B3] Barnett-Cowan M. Harris L. R. (2009). Perceived timing of vestibular stimulation relative to touch, light and sound. Exp. Brain Res. 198, 221–231 10.1007/s00221-009-1779-4 19352639

[B4] Boulinguez P. Nougier V. (1999). Control of goal-directed movements: the contribution of visual attention and motor preparation. Acta Psychol. 103, 21–45 10.1016/S0001-6918(99)00022-0 10555485

[B5] Brenner E. Smeets J. B. J. (1997). Fast responses of the human hand to changes in target position. J. Mot. Behav. 29, 297–310 10.1080/00222899709600017 12453772

[B6] Brenner E. Smeets J. B. J. (2000). Motion extrapolation is not responsible for the flash-lag effect. Vis. Res. 40, 1645–1648 10.1016/S0042-6989(00)00067-5 10814752

[B7] Brenner E. Smeets J. B. J. (2003). Fast corrections of movements with a computer mouse. Spat. Vis 16, 365–376 10.1163/156856803322467581 12858957

[B8] Brenner E. van Beers R. J. Rotman G. Smeets J. B. J. (2006). The role of uncertainty in the systematic spatial mislocalization of moving objects. J. Exp. Psychol. Hum. Percept. Perform. 32, 811–825 10.1037/0096-1523.32.4.811 16846281

[B9] Brière J. Proteau L. (2011). Automatic movement error detection and correction processes in reaching. Exp. Brain Res. 208, 39–50 10.1007/s00221-010-2458-1 20981541

[B10] Cameron B. D. Cheng D. T. Chua R. van Donkelaar P. Binsted G. (2013). Explicit knowledge and real-time action control: anticipating a change does not make us respond more quickly. Exp. Brain Res. 229, 359–372 10.1007/s00221-013-3401-z 23329205

[B11] Cavanagh P. (1997). Visual perception. Predicting the present. Nature 386, 19–21 10.1038/386019a0 9052774

[B12] Chieffi S. Allport D. A. Woodin M. (1999). Hand-centred coding of target location in visuo-spatial working memory. Neuropsychologia 37, 495–502 10.1016/S0028-3932(98)00082-7 10215096

[B13] Cressman E. K. Henriques D. Y. P. (2009). Sensory recalibration of hand position following visuomotor adaptation. J. Neurophysiol. 102, 3505–3518 10.1152/jn.00514.2009 19828727

[B14] Dassonville P. (1995). Haptic localization and the internal representation of the hand in space. Exp. Brain Res. 106, 434–448 10.1007/BF00231066 8983987

[B15] de la Malla C. López-Moliner J. (2012). How timely can our hand movements be? Hum. Mov. Sci. 31, 1103–1117 10.1016/j.humov.2011.12.005 22534212

[B16] Desmurget M. Grafton S. (2000). Forward modeling allows feedback control for fast reaching movements. Trends Cogn. Sci. 4, 423–431 10.1016/S1364-6613(00)01537-0 11058820

[B17] Diedrichsen J. Werner S. Schmidt T. Trommershäuser J. (2004). Immediate spatial distortions of pointing movements induced by visual landmarks. Percept. Psychophys. 66, 89–103 10.3758/BF03194864 15095943

[B18] Elliott D. Hansen S. Mendoza J. Tremblay L. (2004). Learning to optimize speed, accuracy, and energy expenditure: a framework for understanding speed-accuracy relations in goal-directed aiming. J. Mot. Behav. 36, 339–351 10.3200/JMBR.36.3.339-351 15262629

[B19] Engelbrecht S. E. Berthier N. E. O'Sullivan L. P. (2003). The undershoot bias: learning to act optimally under uncertainty. Psychol. Sci. 14, 257–261 10.1111/1467-9280.03431 12741750

[B20] Ernst M. O. Banks M. S. (2002). Humans integrate visual and haptic information in a statistically optimal fashion. Nature 415, 429–433 10.1038/415429a 11807554

[B21] Evarts E. V. Fromm C. (1981). Transcortical reflexes and servo control of movement. Can. J. Physiol. Pharmacol. 59, 757–775 10.1139/y81-112 6459153

[B22] Fetz E. E. Finocchio D. V. Baker M. A. Soso M. J. (1980). Sensory and motor responses of precentral cortex cells during comparable passive and active joint movements. J. Neurophysiol. 43, 1070–1089 6766994 10.1152/jn.1980.43.4.1070

[B23] Flanders M. Cordo P. J. (1989). Kinesthetic and visual control of a bimanual task: specification of direction and amplitude. J. Neurosci. 9, 447–453 2918370 10.1523/JNEUROSCI.09-02-00447.1989 PMC6569788

[B24] Flanders M. Cordo P. J. Anson J. G. (1986). Interaction between visually and kinesthetically triggered voluntary responses. J. Mot. Behav. 4, 427–448 10.1080/00222895.1986.10735389 15138140

[B25] Franklin D. W. Wolpert D. M. (2008). Specificity of reflex adaptation for task-relevant variability. J. Neurosci. 28, 14165–14175 10.1523/JNEUROSCI.4406-08.2008 19109499 PMC2636902

[B26] Georgopoulos A. P. Kalaska J. Massey J. T. (1981). Spatial trajectories and reaction times of aimed movements: effects of practice, uncertainty, and change in target location. J. Neurophysiol. 46, 725–743 7288461 10.1152/jn.1981.46.4.725

[B27] Gritsenko V. Krouchev N. I. Kalaska J. F. (2007). Afferent input, efference copy, signal noise, and biases in perception of joint angle during active versus passive elbow movements. J. Neurophysiol. 3, 1140–1154 10.1152/jn.00162.2007 17615137

[B28] Gritsenko V. Yakovenko S. Kalaska J. F. (2009). Integration of predictive feedforward and sensory feedback signals for online control of visually guided movement. J. Neurophysiol. 102, 914–930 10.1152/jn.91324.2008 19474166

[B29] Harris C. M. Wolpert D. M. (1998). Signal-dependent noise determines motor planning. Nature 394, 780–794 10.1038/29528 9723616

[B30] Johansson R. S. Westling G. (1987). Signals in tactile afferents from the fingers eliciting adaptive motor responses during precision grip. Exp. Brain Res. 66, 141–154 10.1007/BF00236210 3582528

[B31] Khan M. A. Franks I. M. (2000). The effect of practice on component submovements is dependent on visual feedback. J. Mot. Behav. 32, 227–240 10.1080/00222890009601374 10975271

[B32] Krekelberg B. Lappe M. (2001). Neuronal latencies and the position of moving objects. Trends Neurosci. 24, 335–339 10.1016/S0166-2236(00)01795-1 11356505

[B33] Krigolson O. Heath M. (2004). Background visual cues and memory-guided reaching. Hum. Mov. Sci. 23, 861–877 10.1016/j.humov.2004.10.011 15664677

[B34] Lamme V. A. F. (2000). Neural mechanisms of visual awareness: a linking proposition. Brain Mind 1, 385–406 10.1023/A:1011569019782

[B35] Lamme V. A. F. (2003). Why visual attention and awareness are different. Trends Cogn. Sci. 7, 12–18 10.1016/S1364-6613(02)00013-X 12517353

[B36] Lamme V. A. F. Roelfsma P. R. (2000). The distinct modes of vision offered by feedforward and recurrent processing. Trends Neurosci. 23, 571–579 10.1016/S0166-2236(00)01657-X 11074267

[B37] Lamme V. A. F. Supèr H. Spekreijse H. (1998). Feedforward, horizontal, and feedback processing in the visual cortex. Curr. Opin. Neurobiol. 8, 529–535 10.1016/S0959-4388(98)80042-1 9751656

[B38] Linares D. López-Moliner J. Johnston A. (2007). Motion signal and the perceived positions of moving objects. J. Vis. 7, 1–7 10.1167/7.7.1 17685797

[B39] Liu D. Todorov E. (2007). Evidence for flexible sensorimotor strategies predicted by optimal feedback control. J. Neurosci. 27, 9354–9368 10.1523/JNEUROSCI.1110-06.2007 17728449 PMC6673117

[B40] Lönn J. Crenshaw A. G. Djupsjöbacka M. Pedersen J. Johannsson H. (2000). Position sense testing: influence of starting position and type of displacement. Arch. Phys. Med. Rehabil. 81, 592–597 10.1016/S0003-9993(00)90040-6 10807097

[B41] López-Moliner J. Linares D. (2006). The flash-lag effect is reduced when the flash is perceived as a sensory consequence of our action. Vision Res. 46, 2122–2129 10.1016/j.visres.2005.11.016 16405940

[B42] Mima T. Terada K. Maekawa M. Nagamine T. Ikeda A. Shibasaki H. (1996). Somatosensory evoked potentials following proprioceptive stimulation of finger in man. Exp. Brain Res. 111, 233–245 10.1007/BF00227300 8891653

[B43] Nijhawan R. (1994). Motion extrapolation in catching. Nature 370, 256–257 10.1038/370256b0 8035873

[B44] Nijhawan R. (2008). Visual prediction: psychophysics and neurophysiology of compensation for time delays. Behav. Brain Sci. 31, 179–198 discussion: 198–239. 10.1017/S0140525X08003804 18479557

[B45] Nijhawan R. Kirschfeld K. (2003). Analogous mechanisms compensate for neural delays in the sensory and the motor pathways: evidence from motor flash-lag. Curr. Biol. 13, 749–753 10.1016/S0960-9822(03)00248-3 12725732

[B46] Nowak L. G. Munk M. H. J. Girard P. Bullier J. (1995). Visual latencies in areas V1 and V2 of the macaque monkey. Vis. Neurosci. 12, 371–384 10.1017/S095252380000804X 7786857

[B47] Ögmen H. Patel S. S. Bedell H. E. Camuz K. (2004). Differential latencies and the dynamics of the position computation process for moving targets, assessed with the flash-lag effect. Vision Res. 44, 2109–2128 10.1016/j.visres.2004.04.003 15183678

[B48] Oliveira F. T. P. Elliott D. Goodman D. (2005). Energy-minimization bias: compensating for intrinsic influence of energy-minimization mechanisms. Motor Control 9, 101–114 15784952 10.1123/mcj.9.1.101

[B49] Oostwoud-Wijdenes L. Brenner E. Smeets J. B. J. (2011). Fast and fine-tuned corrections when the target of a hand movement is displaced. Exp. Brain Res. 214, 453–462 10.1007/s00221-011-2843-4 21874536 PMC3178780

[B50] Prablanc C. Martin O. (1992). Automatic control during hand reaching at undetected two-dimensional target displacements. J. Neurophysiol. 67, 455–469 1569469 10.1152/jn.1992.67.2.455

[B51] Proteau L. Roujoula A. Messier J. (2009). Evidence for continuous processing of visual information in a manual video-aiming task. J. Mot. Behav. 41, 219–231 10.3200/JMBR.41.3.219-231 19366655

[B52] Raiguel S. E. Lagae L. Gulyàs B. Orban G. A. (1989). Response latencies of visual cells in macaque areas V1, V2 and V5. Brain Res. 1, 155–159 10.1016/0006-8993(89)91010-X 2776003

[B53] Reichenbach A. Thielscher A. Peer A. Bülthoff H. H. Bresciani J. P. (2009). Seeing the hand while reaching speeds up on-line responses to a sudden change in target position. J. Physiol. 19, 4605–4616 10.1113/jphysiol.2009.176362 19675067 PMC2768016

[B54] Rodríguez-Herreros B. López-Moliner J. (2008). The influence of motion signals in hand movements. Exp. Brain Res. 191, 321–329 10.1007/s00221-008-1527-1 18704383

[B55] Rugg M. D. Coles M. G. H. (1995). Electrophysiology of Mind: Event-Related Potentials and Cognition. Oxford: Oxford University Press

[B56] Saunders J. A. Knill D. C. (2003). Humans use continuous visual feedback from the hand to control fast reaching movements. Exp. Brain Res. 152, 341–352 10.1007/s00221-003-1525-2 12904935

[B57] Saunders J. A. Knill D. C. (2004). Visual feedback control of hand movements. J. Neurosci. 24, 3223–3234 10.1523/JNEUROSCI.4319-03.2004 15056701 PMC6730029

[B58] Saunders J. A. Knill D. C. (2005). Humans use continuous visual feedback from the hand to control both the direction and distance of pointing movements. Exp. Brain Res. 162, 458–473 10.1007/s00221-004-2064-1 15754182

[B59] Schiefer U. Strasburger H. Becker S. T. Vontheim R. Schiller J. Dietrich T. J. (2001). Reaction time in automated kinetic perimetry: effects of stimulus luminance, eccentricity, and movement direction. Vision Res. 41, 2157–2164 10.1016/S0042-6989(01)00088-8 11403799

[B60] Seiss E. Hesse C. W. Drane S. Oostenveld R. Wing A. M. Praamstra P. (2002). Proprioception-related evoked potentials: origin and sensitivity to movement parameters. Neuroimage 17, 461–468 10.1006/nimg.2002.1211 12482098

[B61] Smeets J. B. J. van den Dobbelsteen J. J. de Grave D. D. J. van Beers R. J. Brenner E. (2006). Sensory integration does not lead to sensory calibration. Proc. Natl. Acad. Sci. U.S.A. 103, 18781–18786 10.1073/pnas.0607687103 17130453 PMC1693739

[B62] Soechting J. F. Flanders M. (1989). Sensorimotor representations for pointing to targets in three-dimensional space. J. Neurophysiol. 62, 582–594 2769349 10.1152/jn.1989.62.2.582

[B63] Soechting J. F. Lacquaniti F. (1983). Modification of trajectory of a pointing movement in response to a change in target location. J. Neurophysiol. 49, 548–564 6834087 10.1152/jn.1983.49.2.548

[B64] Soso M. J. Fetz E. E. (1980). Responses of identified cells in postcentral cortex of awake monkeys during comparable active and passive joint movements. J. Neurophysiol. 43, 1090–1110 6766995 10.1152/jn.1980.43.4.1090

[B65] Thorpe S. Fize D. Marlot C. (1996). Speed of processing in the human visual system. Nature 381, 520–522 10.1038/381520a0 8632824

[B66] van Beers R. J. Haggard P. Wolpert D. M. (2004). The role of execution noise in movement variability. J. Neurophysiol. 91, 1050–1063 10.1152/jn.00652.2003 14561687

[B67] van Beers R. J. Sittig A. C. Gon J. J. (1999). Integration of proprioceptive and visual position-information: an experimentally supported model. J. Neurophysiol. 81, 1355–1364 10085361 10.1152/jn.1999.81.3.1355

[B68] Veerman M. M. Brenner E. Smeets J. B. J. (2008). The latency for correcting a movement depends on the visual attribute that defines the target. Exp. Brain Res. 187, 219–228 10.1007/s00221-008-1296-x 18256814 PMC2335293

[B69] Veyrat-Masson M. Brière J. Proteau L. (2010). Automaticity of online control processes in manual aiming. J. Vis. 27, 1–14 10.1167/10.14.27 21191135

[B70] Westwood D. A. Heath M. Roy E. A. (2003). No evidence for accurate visuomotor memory: systematic and variable error in memory-guided reaching. J. Mot. Behav. 35, 127–133 10.1080/00222890309602128 12711584

[B71] Whitney D. Murakami I. Cavanagh P. (2000). Illusory spatial offset of a flash relative to a moving stimulus is caused by differential latencies for moving and flashed stimuli. Vision Res. 40, 137–149 10.1016/S0042-6989(99)00166-2 10793892

[B72] Wilson E. T. Wong J. Gribble P. L. (2010). Mapping proprioception across a 2D horizontal workspace. PLoS ONE 5:e11851 10.1371/journal.pone.0011851 20686612 PMC2912297

[B73] Wolpert D. M. Ghahramani Z. Jordan M. I. (1995). An internal model for sensorimotor integration. Science 269, 1880–1882 10.1126/science.7569931 7569931

